# Spotlight on early-career researchers: an interview with Matt Field

**DOI:** 10.1038/s42003-019-0381-y

**Published:** 2019-05-06

**Keywords:** Careers, Lab life, Personalized medicine

## Abstract

Dr. Matt Field is a Senior Research Fellow in Bioinformatics at the Australian Institute of Tropical Health and Medicine at James Cook University, co-director of the Centre for Tropical Bioinformatics and Molecular Biology, and a Chief Investigator for the Centre for Personalised Immunology, an NHMRC Centre of Research Excellence. His research focuses on developing high-throughput bioinformatic analysis pipelines and bringing genomics and personalised medicine into routine clinical practice. In this instalment of our series on early-career researchers, we asked Dr. Field to talk about his research, career trajectory, and the importance of remembering the big picture when working on projects with potential clinical impact.

**Figure:**
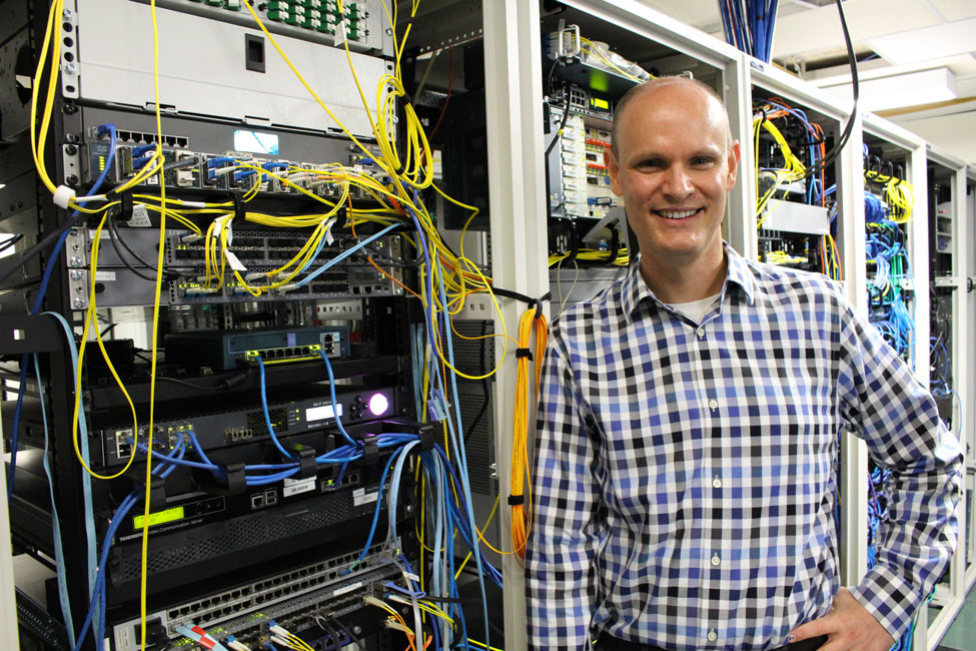
Image credit: Matt Field

Please tell us about your research interests.

My research interest is in developing bioinformatics software and pipelines to uncover the underlying genetic cause of a variety of human diseases. This requires me to develop tools able to analyse large volumes of sequence data and to query increasingly large data sets. A common theme of my research is investigating ways to incorporate cutting-edge sequencing technologies into the health system with a goal of improving health outcomes for individual patients by detecting diseases earlier and more accurately. In addition to human data, I am increasingly looking at pathogen sequence data and the interface between host-pathogen interactions. At the Australian Institute of Tropical Health and Medicine I collaborate with researchers tackling some of the great challenges to health in the tropics, including malaria, dengue fever, tuberculosis and parasitic worms. I also focus on collaborating with clinicians working in tropical health investigating how precision medicine programs can be developed for the treatment of tropical diseases. This is important as 50% of the world’s population is estimated to live in the tropics by 2050.

What has your journey been to this point?

When I first went to university, I followed my interests and studied marine science at the University of British Columbia in Canada. I completed a term of study at a marine research station on Vancouver Island, which was pretty amazing, but I soon realised that while I love biology, I also really enjoy and have a knack for working with large complex data sets. After studying, I travelled for a few years and when I decided to begin my career, I asked my Dad’s academic work colleagues the question: “What skills are most needed in biology now and in the future?” Unequivocally, they responded that there is a growing bottleneck in biological data analysis and that in the future biological data will significantly surpass the means to process it. So, I decided to study computer science and discovered that there was a growing demand for people with the ability to apply computational approaches to solve biological problems. After completing the computer science degree, I worked as a bioinformatician in Canada and later moved to Australia and worked at the Australian National University in a similar capacity. My interest in bioinformatics continued to grow while working for eminent immunologist Chris Goodnow, as he clearly realised the impact bioinformatics was starting to have on medical research. Supported by Chris, I completed a PhD in medical science, during which I developed a high-throughput pipeline to discover the underlying genetic cause of various diseases. After this, I moved to northern Australia where I now develop bioinformatics methods to study tropical diseases at the Australian Institute of Tropical Health and Medicine. Since moving to Cairns, I recognised the growing need for bioinformatics research with a tropical focus and founded and became co-director of the Centre for Tropical Bioinformatics and Molecular Biology. Studying tropical medicine on the doorstep of the Great Barrier Reef also means I have opportunities to pursue my love of marine biology. For example, I just completed a project studying genetic variation in coral. Bioinformatics is a truly dynamic and exciting field: discovering novel ways to utilise bioinformatics to address tropics issues allows me to work across two disciplines I am passionate about.

What are your predictions for your field in the near future?

The field of bioinformatics is dynamic and rapidly changing, and the decrease in the cost of sequencing is rapidly accelerating this shift. Essentially, what used to be slow and cost prohibitive can now be achieved for a fraction of the price and much quicker. Therefore, biology is becoming a data intensive science. Bioinformaticians are working with increasingly large and complex data sets often using several technologies and multi-omic in nature. I predict that the majority of life science students will soon learn bioinformatics as a component of their studies, and it will be common practice for higher degree students to utilise bioinformatics in their research.

Also, bioinformatics will dramatically change how health care is delivered around the world. Treatment will be increasingly personalised and be informed by a patient’s unique genetic sequence, and this will result in tailored treatments designed to improve an individual’s health outcome. Having a genome sequenced will be increasingly widespread and feasible in real time using innovative long read technologies.

Can you speak of any challenges that you have overcome?

While bioinformatics research is now recognised as a stand-alone academic discipline, at the start of my career many biologists did not see the value or need for bioinformatic analysis, which slowed the progress of integrating bioinformaticians into traditional academic positions. For a long time, many academics were reluctant to recognise the value that bioinformatics could add to their research. I was able to overcome this challenge by collaborating with leading researchers who were excited about how bioinformatics could accelerate their research output. Over time these collaborations led to publications that began to demonstrate a growing body of work, and reflected a worldwide trend of increased research output integrating bioinformatics analysis. I still occasionally encounter people who are unsure if bioinformatics can be useful for their discipline but it’s vastly different than at the start of my career.

Another challenge I have overcome early in my career was the tendency to approach problems as one-off analyses that need to be solved as quickly as possible. While this approach worked for a while, I soon realised that many problems have similar patterns and that through proper software design principle and thorough analysis documentation, code could be used over and over again to solve a wide variety of problems. While this approach requires more time up front, overall it resulted in a massive increase in my productivity, which led to promotions. Perhaps the best example of this is the development of a large variant detection pipeline I developed as part of my PhD: I spent a full year designing and implementing infrastructure that focused on the principles of reproducibility, scalability, and flexibility. This pipeline has now been running in a high-throughput research environment for eight years and has recently been approved to run in hospitals as standard practice.

Of course, there have been many more challenges, but those are the ones that stand out.

What advice would you give to your younger self?

I would tell my younger self to ask successful researchers what they wish they knew at the beginning of their career and what they wish they had done differently. Typically, successful academics are genuinely eager to share valuable tips they have learned along the way which can save you a lot of time and energy. Ask them what they believe has contributed to their success and learn from this.

Build a strong academic network of colleagues, including those outside the biological sciences, as increasingly biologists are productively working with chemists, physicists, statisticians, and computer scientists in order to better understand complex biological systems. There are many different ways to problem solve and researchers from other areas often approach a problem from different angles.

What lessons did you learn from your own research, which have shaped your way of thinking?

In my own research it is easy to become overly focused on solving a particular problem. However, working with clinicians has shaped my thinking so that I am now a lot more focused on the end user of the research. Clinicians work with patients every day and want tangible outcomes. So now when I approach a problem, I am not just working with data, but considering how this data might ultimately be used to improve patient health.

Dr. Field’s research website is found at: https://research.jcu.edu.au/portfolio/matt.field/

The Centre for Tropical Bioinformatics and Molecular Biology website is found at: https://www.jcu.edu.au/ctbmb

*This interview was conducted by Associate Editor Jung-Eun Lee*.

